# The impact of maternal HIV infection on cord blood lymphocyte subsets and cytokine profile in exposed non-infected newborns

**DOI:** 10.1186/1471-2334-11-38

**Published:** 2011-02-03

**Authors:** Eliane Borges-Almeida, Helaine MBPM Milanez, Maria Marluce S Vilela, Fernanda GP Cunha, Beatriz M Abramczuk, Suiellen C Reis-Alves, Konradin Metze, Irene Lorand-Metze

**Affiliations:** 1 Department of Internal Medicine, Faculty of Medical Sciences, State University of Campinas, Rua Tessalia Vieira de Camargo 126, 13083-887 - Campinas, Brazil; 2 Department of Gynecology and Obstetrics, Faculty of Medical Sciences, State University of Campinas Av. Alexander Fleming 101, 13083-881 - Campinas, Brazil; 3 Center for Investigation in Pediatrics - CIPED and Department of Pediatrics Faculty of Medical Sciences, State University of Campinas, Rua Tessalia Vieira de Camargo 126, 13083-887 - Campinas, Brazil; 4 Department of Pathology, Faculty of Medical Sciences, State University of Campinas, Rua Tessalia Vieira de Camargo 126, 13083-887 - Campinas, Brazil; 5 Hemocentro - State University of Campinas, Rua Carlos Chagas 480, 13083-878 Campinas - SP Brazil

## Abstract

**Background:**

Children born to HIV+ mothers are exposed intra-utero to several drugs and cytokines that can modify the developing immune system, and influence the newborn's immune response to infections and vaccines. We analyzed the relation between the distribution of cord blood lymphocyte subsets and cytokine profile in term newborns of HIV+ mothers using HAART during pregnancy and compared them to normal newborns.

**Methods:**

In a prospective, controlled study, 36 mother-child pairs from HIV+ mothers and 15 HIV-uninfected mothers were studied. Hematological features and cytokine profiles of mothers at 35 weeks of pregnancy were examined. Maternal and cord lymphocyte subsets as well as B-cell maturation in cord blood were analyzed by flow cytometry. The non-stimulated, as well as BCG- and PHA-stimulated production of IL2, IL4, IL7, IL10, IL12, IFN-γ and TNF-alpha in mononuclear cell cultures from mothers and infants were quantified using ELISA.

**Results:**

After one year follow-up none of the exposed infants became seropositive for HIV. An increase in B lymphocytes, especially the CD19/CD5+ ones, was observed in cord blood of HIV-exposed newborns. Children of HIV+ hard drug using mothers had also an increase of immature B-cells. Cord blood mononuclear cells of HIV-exposed newborns produced less IL-4 and IL-7 and more IL-10 and IFN-γ in culture than those of uninfected mothers. Cytokine values in supernatants were similar in infants and their mothers except for IFN-γ and TNF-alpha that were higher in HIV+ mothers, especially in drug abusing ones. Cord blood CD19/CD5+ lymphocytes showed a positive correlation with cord IL-7 and IL-10. A higher maternal age and smoking was associated with a decrease of cord blood CD4+ cells.

**Conclusions:**

in uninfected infants born to HIV+ women, several immunological abnormalities were found, related to the residual maternal immune changes induced by the HIV infection and those associated with antiretroviral treatment. Maternal smoking was associated to changes in cord CD3/CD4 lymphocytes and maternal hard drug abuse was associated with more pronounced changes in the cord B cell line.

## Background

HIV infection is associated with a complex pattern of changes in the hemopoietic and the immune systems, resulting in abnormalities of peripheral blood (PB) counts and changes in T and B lymphocytes. Decrease of T helper and increase of cytotoxic lymphocytes, profound changes in the cytokine profile and a variety of B lymphocyte abnormalities have been repeatedly described [1-4]. However, long term antiretroviral therapy (ARV) is able to restore, at least in part, the immune function [3-6].

The introduction of ARV therapy in HIV+ pregnant women has drastically decreased vertical transmission of HIV [1-7]. But, several changes in PB counts and T CD4^+ ^and CD8^+ ^lymphocytes have been detected in HIV-exposed uninfected newborns [1,2,4] and attributed to alterations in maternal cytokine profile caused by the HIV infection as well as by the ARV treatment [2,8-14]. PB count changes are soon reversed, but some T lymphocyte changes may last for as long as 8 years [2]. Therefore, these infants present an increased risk for severe infections. This risk is further increased as newborns from HIV-infected mothers usually do not receive breast feeding in order to avoid vertical transmission. Changes in T lymphocytes may also affect the response to vaccines given in the neonatal period [2,10-13].

Alterations in infants' T lymphocyte subsets have been well studied, but little is known about the impact of HIV infection and highly active antiretroviral treatment (HAART) on neonatal maturation and function of B lymphocytes [8,15].

The aim of our study was to analyze the B cell maturation in umbilical cord blood of infants born to HIV-infected mothers using HAART. We also studied the relation between the distribution of lymphocyte subsets and cytokine production in short term cultures of cord blood mononuclear cells in as well as in maternal peripheral blood mononuclear cells at 35 weeks of pregnancy. We also looked for the relation between maternal smoking and use of hard drugs during pregnancy and infant's lymphocyte subpopulations.

## Methods

### Mother-child pairs

We studied 36 mother-child pairs of HIV positive pregnant women attended at our High Risk Obstetric Unit. They were >18 years old, were using HAART during gestation and had a low or undetectable viral load. All of them had a term delivery. None of the newborns had a malformation at birth. Their data were compared to 15 normal mother-child pairs, which were also attended at our Institution. Mothers of the control group were also >18 years old, had no known pathological condition: hypertension, diabetes, obesity, autoimmune disorders, infections, nor a past history of repeated infections suggesting an underlying immunodeficiency, and had a normal term delivery.

Peripheral blood counts as well as cytokine profile of both groups of mothers as well as viral load of the HIV+ ones were taken between 32 to 35 weeks of gestation.

After 13-17 months of observation none of the exposed infants developed HIV infection. Infants were considered HIV- uninfected and defined as seroreverters if they had negative HIV- polymerase chain reaction (PCR) tests at 1 and 3 months of age or became HIV-seronegative after 13 months of age.

### Cord blood analysis

**Figure 1:**
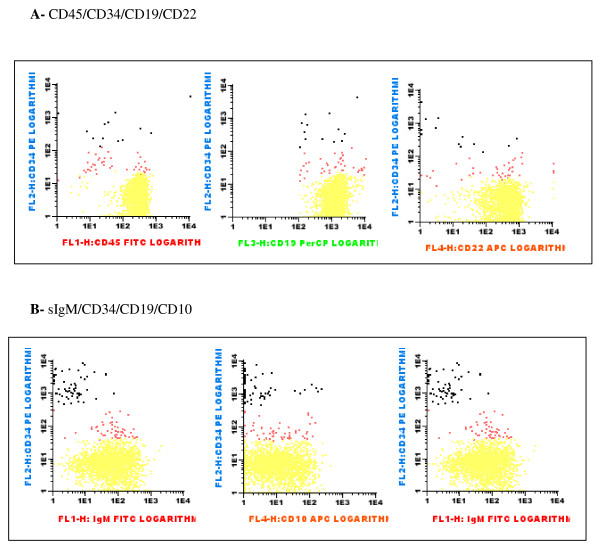
**Analysis of B-cell maturation in cord blood of a normal newborn (control group)**. Expression of CD34 was used to identify immature B-cells. Maturation was studied using the expression of CD45, CD22, CD10 and membrane IgM (sIgM). A) CD45/CD34/CD19/CD22 combination - black: immature cells - CD45^low^CD34^+^CD19^+^CD22^- ^red: intermediate cells - CD45^-/+^CD34^low^CD19^+^CD22^+ ^yellow: mature cells - CD45^+^CD34^-^CD19^+^CD22^+^. B) sIgM/CD34/CD19/CD10 combination - black: immature cells - sIgM^-^CD34^+^CD19^+^CD10^-/+ ^red: intermediate cells - sIgM^+^CD34^-/+^CD19^+^CD10^-/+ ^yellow: mature cells - sIgM^+^CD34^-^CD19^+^CD10^-/+^.

Cord blood was separated at delivery and processed during the first 24 hours after collection. Cell counts were performed in the hematologic counter Advia 120 (Bayer, Dublin - Ireland). Lymphocyte subsets were studied by flow cytometry. T lymphocytes were analyzed in the CD4/CD8/CD3 combination. Their number was expressed as percentage of CD3+ cells among all cord blood nucleated cells. B lymphopoiesis was studied in the combinations: CD5/CD19/CD45, CD45/CD34/CD19/CD22 and sIgM/CD34/CD19/CD10 (Figure 1). For each case, at least 50000 events were acquired using the FACSCalibur (Beckton Dickinson) equipment (Cell Quest software). Analysis was performed using the Paint-a-Gate software.

### Cytokine Assays

Cytokine assays were performed in supernatants from cultures of peripheral blood mononuclear cells (PBMC) of the mothers at 35 weeks of gestation, and infants' cord blood mononuclear cells (CBMC). In both cases, mononuclear cells were isolated by Ficoll-Hypaque 1077 (Sigma, MO, USA). They were suspended in RPMI 1640 with 10% human serum, 1% glutamine and 0.1% gentamicine and cultured for 48 hours with live Bacillus Calmette-Guérin (BCG) (5 × 10^5^/mL), (Butantan Institute, SP), phytohemagglutinin (PHA, 7.5 μg/mL) (Sigma, MO, USA) or in medium alone (non-stimulated).

Cytokines were quantified by enzyme-linked immunosorbent assay (ELISA) in cell-free supernatants using commercial kits for human IL-2, IL-4, IL-7, IL-10, IL-12, TNF-α and IFN-γ (Duo Set^®^, R&D Systems Inc, Minneapolis, MN, USA). These cytokines were chosen as they are able to analyze Th1 and Th2 status [15].

### T-cell proliferation

**Figure 2:**
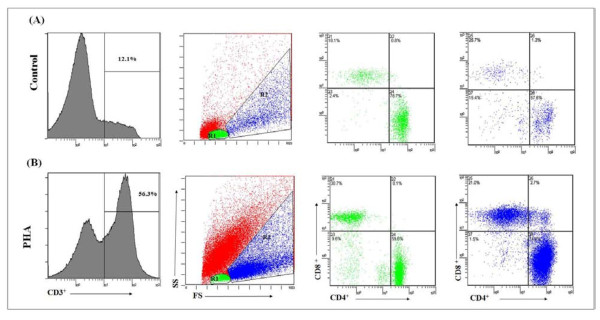
**Analysis of T cell proliferation of non-stimulated (A) and PHA-stimulated (B) cord mononuclear cells of a newborn from a HIV-infected mother**. After gating of CD3 cells, events were analyzed for size and complexity (forward-scatter and side-scatter gates), from which resting (R1-green) and blast cells (R2-blue) were separated. T-lymphocyte subsets were then analyzed. Dead cells (red) were excluded from all analyses. T cell proliferation was the difference between the percentages of PHA-stimulated blasts and non-stimulated blasts.

Cord Blood mononuclear cells (CBMC) were isolated by density gradient centrifugation over Ficoll-Hypaque (Amersham Biosciences, USA), washed, diluted to 1 × 10^6 ^cells/mL in RPMI 1640 medium (Sigma, USA), supplemented with 10% human AB serum (Sigma, USA), 1% glutamine (Sigma, USA) and 0.1% gentamycin and stimulated for 6 days with PHA or medium alone at 37°C with 5% CO2 96-well tissue culture plates (NUNC, Denmark). After harvesting in 20 mM EDTA, samples were incubated with human immunoglobulin and then stained with anti-human CD3, CD4 and CD8 (Beckman Coulter, USA) fluorescent antibodies before acquisition and analysis. Only CD3+ T cells were used in the analysis. Resting and blast lymphocytes were gated on the forward and side scatter plot (Figure 2). Dead cells were excluded from all analyses. CD4+ and CD8+ cells were identified in the gate of blast lymphocytes. Proliferation was measured as percentage of CD3+ blasts in the PHA-stimulated well from which basal proliferation without stimulation was subtracted (PHA, Sigma, USA, 7.5 μg/mL).

### Statistical Analysis

Features of the normal and HIV-exposed newborns and their mothers were compared by the Mann-Whitney and Kruskall-Wallis tests. Values were considered statistically significant when the two-sided p-value was < 0.05. The relationships among the features were examined by the Spearman rank order correlation test. In order to find out which variables were related to birth weight and the T and B cell subsets, multiple step-wise linear regressions were run (0.05 for input and p = 0.1 for output, backward conditional stepwise selection) using the variables showing significant differences or correlations in the univariate analysis. Moreover, the R2 values of each regression, which represent the goodness-of-fit of the mathematical model, were recorded in order to estimate the approximation of the algorithm to clinical and biological reality. Finally, the stability of the model was tested by bootstrap resampling. In this technique we create new data sets of equal size by random sampling of the original data with replacement [16-18]. Thus, in a new bootstrap sample, a patient may be represented once, multiple times or not at all. Then, for each of these "new" sets, a linear regression is calculated and the variables entering each of the "new" models are recorded. This procedure tests the stability of a mathematical model, points out the most important variables and, furthermore, permits to calculate confidence intervals (IC). WinStat 3.1 software was used.

### Ethics Approval

All mothers were invited to participate, received a complete explanation about the aims of the study and written informed consent was obtained according to our local Ethics's Committees (CAISM - Center of Integrated Care for Women's Health, and that of our Faculty of Medicine).

## Results

### Pregnant women

The epidemiological features of the mothers entering the study are presented in Table 1. In the control group, only one mother was a smoker. Yet, smoking and drug abuse (marihuana, cocaine or crack) was common among the HIV+ ones. Both maternal groups had a similar age and a similar time of gestation. HIV+ mothers had a cesarean section in all but one case. Among HIV-negative mothers 11 had a vaginal and 4 a cesarean delivery. Antiretroviral therapy included protease inhibitors in all but 3 cases. Mean time of maternal HAART treatment was 5 months. Eight mothers got pregnant while already using HAART. HIV viral load was undetectable in 23 HIV+ mothers and low in the remaining thirteen (Table 1).

**Table 1:** Epidemiologic characteristics of the mothers (median and range)

	HIV negative (controls)	HIV+ mothers
Age at delivery (years)	29 (18 - 41)	32 (20 - 39)
Weeks of gestation	39.7 (36 - 42)	38.2 (36 - 42)
Smoker only	1	5
Drug user only	0	3
Smoker and drug user	0	6
Non-smoker non-drug user	14	22
Days of HAART use	-	154 (49 - 273)
detectable viral load copies/mL*	-	496 (54 - 36586)

* among 13 mothers with a detectable viral load.

Mothers' PB counts are shown on Table 2. HIV+ mothers had hemoglobin values similar to that of HIV-uninfected ones, but showed a significantly higher mean corpuscular volume (MCV), lower total leukocyte and neutrophil counts. The proportion of T CD4+ and T CD8+ lymphocytes of the HIV+ mothers presented similar values in smokers, non-smokers and drug users.

**Table 2:** Peripheral blood features of the mothers at 35 weeks of pregnancy (median and range)

	HIV negative mothers	HIV+ mothers	p value
RBC* (M/μL)	3.9	3.3	<0.005
	(3.7 - 4.7)	(2.5 - 4.7)	
HB* (g/dL)	11.7	10.7	0.09
	(9.6 - 13.9)	(9.0 - 13.7)	
MCV* (fL)	88	104	<0.005
	(72 - 94)	(75 - 118)	
WBC* (K/μL)	8.2	7.2	0.04
	(3.2 - 12.2)	(3.0 - 15.0)	
Total neutrophils (K/μL)	5.7	4.86	0.07
	(2.3 - 8.8)	(2.2 - 10.9)	
Total lymphocytes (K/μL)	1.8	1.6	0.94
	(0.6 - 2.7)	(0.5 - 4.8)	
CD4+ count	n.d.*	511	
		(57 - 1189)	
CD8+ count	n.d.*	676	
		(321 - 1979)	
CD4/CD8 ratio	n.d.*	0.73	
		(0.06 - 1.97)	
Platelets (K/μL)	200	217	0.96
	(102 - 392)	(99 - 360)	

* RBC = red blood cells; HB = hemoglobin; MCV = mean corpuscular volume; WBC = leukocytes; n.d = not done

The cytokine concentrations in the supernatant of PBMC cultures not stimulated and after stimulation with BCG and PHA in mothers at 35 weeks of gestation are presented on Table 3. HIV+ mothers showed lower values of IL-4 and IL-7, but higher values of TNFα in non-stimulated cultures. Production of IL-2 and IL-12 by PBMCs in non-stimulated as well as stimulated cultures were similar both in normal and HIV-infected mothers.

**Table 3:** Cytokine levels (in pg/ml, median and range) without antigenic stimulus and after stimulation with BCG and PHA in the supernatant from mothers' peripheral mononuclear cell cultures collected at 35 weeks of gestation

	HIV-uninfected controls	HIV+ mothers	p value
IL-2 non-stimulated	0.0 (0 -76)	0.0 (0 - 767)	0.47
BCG	0.0 (0 - 78)	2.14 (0 - 232)	0.44
PHA	89.8 (0 -968)	24.5 (0 - 1421)	0.34
IL-4 non-stimulated	41.0 (38 - 278)	22.2 (9.2 - 76)	<0.005
BCG	39.1 (37 - 216)	22.8 (9.5 - 76)	<0.005
PHA	52.0 (39 - 146)	30.14 (14 - 419)	<0.005
IL-7 non-stimulated	67.0 (0 -88)	0.0 (0 - 90)	0.05
BCG	66.0 (0 - 89)	0.0 (0 - 86)	0.04
PHA	66.0 (0 - 85)	0.0 (0 - 87)	0.03
IL-10 non-stimulated	0.0 (0 - 490)	17 (0 - 18015)	0.14
BCG	736 (0 - 4910)	764 (0 - 43384)	0.49
PHA	498 (0 - 2925)	405 (0 - 58344)	0.56
IL-12 non-stimulated	0.51 (0 - 2.83)	0.51 (0 -2.4)	0.62
BCG	1.18 (1 - 31)	1.0 (0 - 11)	0.27
PHA	1.34 (0 - 51)	1.53 (0 - 33)	0.68
IFNγ non-stimulated	0.0 (0 - 94)	13.2 (0 - 131)	0.73
BCG	2.73 (0 - 1343)	29.2 (0 - 999)	0.42
PHA	110.2 (0 - 2528)	308.9 (0 - 5143)	0.16
TNFα non-stimulated	245.4 (0 - 4826)	526.8 (0 - 7928)	<0.0005
BCG	5374.0 (0 - 13852)	2072.7 (86 - 34130)	0.83
PHA	827.6 (261 - 20889)	1513.3 (244 - 14448)	0.22

The CD4/CD8 ratio in PB of HIV+ mothers showed an inverse relation with viral load (r = -0.30; p = 0.02) and production of TNFα by PBMCs (non-stimulated) (r = -0.27; p = 0.04) and a direct correlation with IL-10 (r = 0.35; p = 0.01). There was no correlation with IL-7.

In a multiple regression, we obtained the formula:

Maternal CD4/CD8 ratio = 0.946 + 5.6 × 10^-5 ^× IL-10 - 9.8 × 10^-5 ^× TNFα - 0.0567 × log viral load + 0.9468 R^2 ^= 0.335 ; p = 0.004

The stability of the model was confirmed in a bootstrap resampling procedure. Among 100 new models, "log viral load" entered in 97%, "IL-10" level in 89%, "TNFα" in 60% and "IL-7" in 28% of them.

### Newborns

The birth data of the newborns are described in Table 4. Children of HIV+ mothers had a similar height but a lower weight than those born to uninfected mothers. Furthermore, they had lower hemoglobin levels, a higher MCV and lower leukocyte and neutrophil counts than non-exposed newborns. The number of days of maternal ARV treatment during pregnancy did not influence these values, but there was a good correlation between maternal and cord blood red blood cells (RBCs) (r = 0.56; p < 0.0005) and MCV (r = 0.54; p < 0.0005). This was less evident concerning hemoglobin values (r = 0.21; p = 0.06).

**Table 4:** Birth data, cord blood counts and lymphocyte subsets of the newborns (median and range)

	Not exposed (controls)	HIV - exposed	p value
Weight (grams)	3100	2962	<0.005
	(2988 - 3915)	(1885 - 3885)	
Height (cm)	48	48	0.87
	(40 - 53)	(33 - 53)	
Capurro score	278	267	<0.005
	(256 - 294)	(255 - 293)	
RBC (M/μL)	4.6	3.5	<0.005
	(4.1 - 5.0)	(2.7 - 4.7)	
HB (g/dL)	15.7	13.7	<0.005
	(13.2 - 18.0)	(9.1 - 16.4)	
MCV (fL)	104	117	<0.005
	(99 - 124)	(88 - 150)	
Platelets (K/μL)	202	242	0.12
	(119 - 328)	(103 - 455)	
WBC (K/μL)	11.1	9.1	0.08
	(4.4 - 19.5)	(4.0 - 22.0)	
Total neutrophils (K/μL)	4.2	3.5	0.32
	(0.8 - 9.4)	(1.0 - 8.8)	
Total lymphocytes (K/μL)	5.7	5.1	0.16
	(3.0-9.1)	(1.5 - 10.5)	
% CD3+	18.3	24.8	0.03
	(13.8 - 32.3)	(12.0 - 41.5)	
% CD3/CD4+	12.6	17.2	0.32
	(8.9 - 23.3)	(3.0 - 28.2)	
% CD3+/CD8+	4.6	6.4	0.002
	(1.8 - 10.7)	(2.3 - 15.5)	
CD4/CD8 ratio	3.5	2.3	0.04
	(1.6 - 8.3)	(0.2 - 6.5)	
% CD19+	3.6	5.7	0.01
	(2.0 - 5.9)	(1.7 - 16.2)	
% CD19+CD5+ **	59.5	75.8	0.006
	(42.8 - 91.1)	(47.7 - 97.0)	

* RBC = red blood cells; HB = hemoglobin; MCV = mean corpuscular volume; WBC = leukocytes, ** among all CD19+ cells

The birth weight of HIV-exposed infants showed a relation with the duration of maternal HAART treatment during pregnancy and the TNFα production (non-stimulated cultures) in supernatants of cultures of PB mononuclear cells collected at the end of gestation. Maternal age, smoking, weeks of gestation and child's gender had no relation to birth weight.

In a multiple regression we obtained the formula:

Birth weight = 3327 - 1.48 × days of HAART - 5 × unstimulated maternal TNFα p = 0.006, R^2 ^= 0.216 ( CI 95% : 0.215 - 0.383 )

The bootstrap resampling study confirmed this result: "days of HAART treatment" appeared in 77% and "non-stimulated maternal TNFα at 35 weeks of gestation" was present in 70% of the new data sets. But, "weeks of gestation" appeared in 40%, "maternal smoking" in 38%, "mother's age" in 12% and "child's gender" in 10% of the new data sets.

The distribution of the several lymphocyte subsets in cord blood is shown on Table 4. The percentage of CD3+ lymphocytes in HIV-exposed newborns was increased when compared with the non-exposed ones. The CD4/CD8 ratio was significantly lower.

In HIV-exposed neonates the relation of the number of cord T lymphocytes with maternal age, days of HAART use during pregnancy, smoking and drug abuse was examined in a multiple regression. For T CD8+ cell no model could be obtained.

The percentage of T CD4+ cells was lower in children from older and smoking mothers.

This percentage could be calculated by the formula:

Cord % T CD4+ cells = -0.428 × mother's age (years) - 4.91 (S) + 30.52;

S = 1 for smoking and 0 for non-smoking mother.

R^2 ^= 0.242 (CI 95%: 16.6 - 51.1)

Drug abuse and days of maternal HAART treatment during pregnancy did not enter the model. The stability of the model for cord blood T CD4+ cells was confirmed in the bootstrap resampling. Among 100 new models, "maternal age" entered in 87% and "maternal smoking" in 72% of them. "Duration of maternal HAART treatment" entered in 42%, and "drug abuse" in 11% of the models.

CD3, CD4 and CD8 immunophenotyping was carried out in cell cultures of cord blood from 22 newborns from HIV + mothers and 10 non-exposed ones. Median background CD3+ proliferation was 9.6% (0.5-25.9) in the HIV+ group and 10.4% (6.2-91.7) in the controls (p = 0.44). The blast percentage in PHA-stimulated cultures showed a median of 40.3% (1.5-71.6) for the HIV+ group and 54.2 (11.7-74.8) for the non-exposed controls. The proportion of PHA-proliferating CD4+ and CD8+ cells were not different between the groups (Table 5).

**Table 5:** Percentages of CD4+ and CD8+ blastic cells determined by flow cytometry in CBMC^a ^cultures incubated with PHA^b ^from 22 newborns from HIV+ mothers and 10 HIV negative controls (median and range)

T cells	Non exposed controls	HIV exposed	p value
CD4+	88.6	86.3	0.807
	(3.4-97.2)	(1.7-98.3)	
CD8+	6.2	7.6	1.00
	(1.4-79.5)	(0.3-92.7)	

^a ^cord blood mononuclear cells; ^b^Phytohemaglutinin

Cord blood CD19+ lymphocytes (Table 6) were increased in newborns of HIV+ mothers due to an increase of CD19+/CD5+ cells. Children of drug users had the highest numbers of total B lymphocytes. The duration of HAART during pregnancy showed no correlation with the total number of cord blood B-cells or with that of CD19+/CD5+ ones.

**Table 6:** B lymphocyte subsets and B-cell maturation in cord blood studied in the CD45/CD34/CD19/CD22 and sIgM/CD34/CD19/CD10 combinations (median and range)

	Non exposed controls	HIV exposed	HIV exposed drug using mother	p value
% CD19^+^	3.6	5.2	7.0	0.01
% CD19^+^CD5^+ ^*	59.5	77.8	67.6	0.007
% CD45^low^CD34^+^CD19^+^CD22^-/+ ^**	0.73	0.42	1.1	
	(0.2 - 3.13)	(0.07 - 1.22)	(0.78 - 1.4)	0.01
% CD45^-/+^CD34^low^CD19^+^CD22^+ ^***	2.62	2.94	13.0	
	(0.24 - 6.8)	(1.5 - 8.2)	(8.8-19.4)	0.01
% CD45^+^CD34^-^CD19^+^CD22^+ ^****	96.7	96.7	86.3	
	(91 - 99)	(91.6 - 98.4)	(79 - 90)	0.02
%sIgM^-^CD34^+^CD19^+^CD10^-/+ ^**	0.4	0.2	1.5	
	(0.10-2.2)	(0.05-0.95)	(0.4-2.7)	0.05
%sIgM^+^CD34^-/+^CD19^+^CD10^-/+ ^***	2.4	2.2	6.5	
	(0.87-7.1)	(0.6-4.5)	(2.1-71)	0.13
%sIgM^+^CD34^-^CD19^+^CD10^-/+ ^****	97.1	97.3	92.5	
	(91.3-98.9)	(94.6-99.3)	(92.0-95.0)	0.05

* percentage of the total CD19^+ ^cells, ** immature cells, ***intermediary and ****mature B-cells

B-cell maturation was analyzed in two four-color combinations (Table 6). The CD45/CD34/CD19/CD22 combination was the most useful to reveal an increase in immature and intermediary B-precursors in infants of HIV+ mothers. This feature was more pronounced in children of drug users.

In HIV-exposed neonates, there was no significant difference in the proportion of immature cells with the newborn's gender (p = 0.33) or children of smokers and non-smokers (p = 0.21), but those of drug users showed a higher percentage (p = 0.007) (Table 6). There was no significant correlation between this variable and gestation duration or maternal age but with the number of days of maternal HAART use (r = 0.53; p = 0.01).

In a multiple regression, examining the relation between the percentage of cord blood immature cells with maternal drug abuse and the number of days of HAART treatment during pregnancy, the proportion of immature/intermediate (IMAT) B cells could be calculated by the formula:

% Cord IMAT B cells = 0.02 × days of HAART + B + 0.72; R^2 ^= 0.859 (CI - 0.653 - 0.943)

With B = 12.0 for drug abusers and B = 0 for all other mothers.

The stability of this regression model was tested by the bootstrap resampling study, where both variables entered in 98% of the new data sets.

The cytokine concentrations in the supernatant of CBMC cultures (Table 7) showed a high correlation with the values found for their mothers (Table 3), excluding those of IFNγ and TNFα. IL-4 and IL-7 production was lower in HIV+ mothers and cord blood mononuclear cells. Concerning IL-10, there was no significant difference between HIV+ and HIV negative mothers. However, HIV-exposed newborns produced higher amounts than non-exposed, although these differences had a low statistical significance.

**Table 7:** Cytokine levels (in pg/ml, median and range) without antigenic stimulus and after stimulation with BCG and PHA in the supernatant of cord blood cell cultures

	Non-exposed newborns	HIV-exposed newborns	p value
IL-2 non-stimulated	0.0 (0 -76)	0.72 (0 - 81)	0.13
BCG	0.0 (0 -78)	0.36 (0 - 79)	0.54
PHA	43.7 (0 - 1278)	18.9 (0 - 5936)	0.46
IL-4 non-stimulated	39.2 (39 - 76)	25.5 (10 - 76)	<0.005
BCG	39.4 (40 - 76)	25.6 (9 - 76)	<0.005
PHA	39.2 (39 - 78)	26.7 (9.5 - 76)	<0.005
IL-7 non-stimulated	66.0 (0 - 87)	0.0 (0 - 87)	0.13
BCG	66.4 (0 - 87)	1.5 (0 - 93)	0.24
PHA	66.5 (0 - 87)	0.0 (0.0 - 88)	0.11
IL-10 non-stimulated	0 (0 - 88)	3.5 (0 - 1028)	0.13
BCG	199 (0 - 1522)	582 (0 - 7049)	0.06
PHA	224 (0 - 1934)	410 (0 - 36093)	0.19
IL-12 non-stimulated	0.66 (0 - 13.3)	0.24 (0 - 5.7)	0.41
BCG	0.83 (0 - 26)	0.74 (0 - 3.64)	0.10
PHA	0.62 (0 - 3)	1.1 (0 - 8)	0.58
IFNγ non-stimulated	0.0 (0 - 66)	17.5 (0 - 194)	0.01
BCG	0.0 (0 - 1188)	19.4 (0 - 550)	0.02
PHA	24.3 (0 - 2445)	73.2 (0 - 2928)	0.28
TNFα non-stimulated	136.4 (0 - 500)	181.6 (0 - 3381)	0.66
BCG	1458.1 (138 - 7026)	1069.0 (283 -14724)	0.96
PHA	535.0 (298 - 5672)	658.4 (0 - 6313)	0.63

Concerning TNFα, HIV+ mothers had a higher production, especially drug users (p = 0.01). CBMCs of HIV-exposed infants produced similar amounts of TNFα but more IFNγ than the control group. No correlation was found between the number of days of maternal HAART treatment during pregnancy and CBMC cytokine production.

In non-exposed newborns, no correlation was found between the percentage of CD19+ cells as well as CD19+/CD5+ ones and any cytokine production in CBMCs. However, in HIV-exposed newborns, an inverse correlation was found between total cord blood CD19+ cells and cord IL-4 and IL-7 production in non-stimulated cultures (Table 8).

**Table 8:** Correlations found in HIV-exposed neonates between cord blood B-cell subsets and cytokine production by cord mononuclear cells in culture

	CD19+ cells	CD19+/CD5+ cells
IL-4 non-stimulated	r = - 0.49; p = 0.001	r = 0.25; p = 0.07
BCG	r = - 0.46; p = 0.002	n.s.*
PHA	r = - 0.40; p = 0.008	n.s.*
IL-7 non-stimulated	r = - 0.46; p = 0.002	r = 0.32; p = 0.02
BCG	r = - 0.39; p = 0.01	r = 0.41; p = 0.006
PHA	r = - 0.48; p = 0.001	r = 0.45; p = 0.003
IL-10 non-stimulated	r = - 0.31; p = 0.05**	r = 0.33; p = 0.02
BCG	r = - 0.48; p = 0.02**	r = 0.51; p < 0.0005
PHA	r = - 0.39; p = 0.04**	r = 0.41; p = 0.03

* not significant; ** considering only immature/intermediary B-cells

A positive correlation was seen between CD19+/CD5+ cells and cord IL-7 and IL-10, but no correlation was found between these cells and production of IL-4, IFNγ and TNFα by CBMCs.

## Discussion

HIV-exposed uninfected infants present a variety of small but significant alterations compared to non-exposed neonates such as a lower birth weight, peripheral blood cytopenias and distribution pattern of circulating lymphocyte subsets [1,2,4,5,10,19-25]. Their lower birth weight has been attributed to an earlier delivery due to a higher rate of elective cesarean section [10] or to an earlier maternal Th2 to Th1 shift related to the antretroviral treatment leading to a premature delivery [15]. In our study however, the gestation period was similar in both groups despite that HIV+ mothers delivered by an elective cesarean section. Low weight at birth of HIV-exposed infants was associated to a high maternal production of TNFα at the end of pregnancy, which was more common in drug abusing mothers. It has been shown that neither HAART nor decreased IL-10 levels, but only increase in maternal IL-2 is associated to a premature delivery [26-28]. To the best of our knowledge there are no data in the literature about the relation between maternal IL-7 and IL-4 and low birth weight and prematurity. In our study, a better control of the maternal HIV infection, expressed by an increase of the CD4/CD8 ratio, was also associated with a lower production of TNFα and higher one of IL-10.

In our prospective controlled trial we compared hematological and immunological features of term newborns from HIV+ women under effective HAART treatment during pregnancy with normal unexposed newborns. Hemoglobin, leukocyte and neutrophil counts were lower, and MCV was higher. These alterations were similar to those described in the literature [1,2,4,10], and could be readily explained by maternal HAART treatment and placental drug transfer, as it has been already pointed out [4,25]. We could not demonstrate an association of the intensity of the newborns' peripheral cytopenias with the duration of maternal HAART treatment, but when maternal and newborns' PB counts were compared, RBC and MCV showed a significant correlation. We could not find any correlation concerning leukocytes and platelets. Our study cohort was rather small, but other factors, such as maternal nutritional status or use of antibacterial medications could have influenced the PB counts found.

We could also detect several differences in cord blood lymphocyte sub-populations in our HIV-exposed uninfected newborns when compared to children from HIV negative mothers. We studied the possible association of these alterations with the maternal and cord blood cytokine profile, duration of maternal HAART treatment as well as maternal smoking and drug abuse.

Concerning maternal cytokine profile, HIV+ women only differed from uninfected ones by a lower production of IL-4 and IL-7 in culture, but more TNFα, especially in drug using women. A decrease in the Th2 cytokine profile has been described at the end of pregnancy [26]. On the other hand, antiretroviral drugs, especially protease inhibitors as used in the present study, have been associated to a metabolic syndrome associated with an activation of pro-inflammatory cytokines, mainly TNFα [27]. We may speculate that the co-occurrence of maternal HIV-1 infection, use of HAART and hard drugs, all contributed to a higher stimulation of maternal immune cells leading to a larger baseline production of TNF-α in short term culture. The reduced production of IL-4 and IL-7 observed in HIV-infected mothers is in keeping with a change in T-lymphocyte homeostasis in HIV-infected patients.

The decrease of IL-4 and increase of IFN-γ in HIV-exposed uninfected newborns speaks in favor of an environment with a Th1-shifted balance. This is more pronounced in newborns of HIV+ mothers under effective HAART [4,6,11,12]. This treatment has been associated with an at least a partial restoration of the immune function in HIV infected children and adult patients [19-22].

Our results confirmed previous findings of changes in T-cell development in cord blood of uninfected newborns of HIV - infected mothers with undetectable or low viral load [2,9-11]. Our HIV-exposed uninfected newborns presented higher numbers of CD3^+ ^cells, due mainly to increase in CD8^+ ^lymphocytes as is seen in HIV-infected adults. Lower proportions of CD4^+^, but not of CD8^+ ^T cells was also associated to a higher maternal age and smoking. Although we examined only a small number of patients, these findings are in keeping with those found in adult smokers [29,30]. Therefore, not only the maternal HIV infection could alter the thymus function of exposed infants, but also maternal smoking.

Placental transfer of HIV-related proteins and residual maternal immune alterations could be responsible for the findings concerning the immune function of our HIV-exposed non-infected neonates [31,32]. On the other hand, 11/15 of the healthy control mothers in our study had a normal vaginal delivery, while all except one of the HIV-infected mothers had a cesarean section. Therefore, some differences found between HIV-exposed and non-exposed neonates may be explained by the different mode of delivery of both groups of newborns. It has been shown that normal labor induces immune stimulation in mothers and neonates including a higher production of TNF-α [33]. This could explain the difference found of this cytokine in supernatants of maternal but not in cord mononuclear cultures, as normal newborns were stimulated by labor and HIV-exposed ones were already stimulated by the maternal HIV infection.

The long term CBMC cultures demonstrated similar numbers of T-derived activated cells after PHA stimulation in HIV-exposed neonates and non-exposed ones. Similar proportions of CD4+ and CD8+ cells were also observed in HIV-exposed uninfected newborns and unexposed ones, speaking in favor of a preserved function of these cells.

Cord blood B lymphocytes were increased in HIV-exposed neonates, mainly caused by an increase in CD19+/CD5+ cells. A B-cell dysfunction with appearance of immature/transitional B-cells and hypergammaglobulinemia has also been described in active HIV infection in adults [8,20,34-36]. There are some discrepancies in the literature concerning the change in number of these cells in adult HIV-infected patients [19-24]. However, long-term response to HAART seems to restore at least partially, the number and function of these cells [8,10,12,19,20]. In the present study, the number of CD5+ B lymphocytes was correlated directly with production of IL-7 and IL-10 by CBMCs. CD5 is a negative regulator of the B-cell receptor signaling contributing to a prolonged cell survival and maintaining tolerance in anergic B cells in vivo [37]. In cord blood, CD5+ B lymphocytes represent about half of the B cells [38]. The increase of CD5+ B lymphocytes in our HIV-exposed newborns may be an expression of an immune deregulation due to placental transfer of viral proteins and cytokines. How these changes may affect the neonatal development of HIV-exposed uninfected children is not known, but a reduced response to neonatal vaccines has been reported [13]. So, the changes found in our HIV-exposed newborns indicate that, although HAART controlled maternal HIV infection, leading to low or undetectable viral load at 35 weeks of pregnancy, children still had changes in B lymphocytes, consistent with some remaining HIV-mediated immune abnormalities.

We studied B-cell maturation in cord blood using four-color-antibody combinations proposed by van Lochen et al [39]. Only the combinations that examined the initial maturation of the B cell line were able to disclose differences between normal newborns and those born to HIV+ mothers. Especially maternal drug abuse and a longer period of maternal HAART treatment during pregnancy were associated with a larger number of immature B-cells. This could be a sign of a higher fetal burden of HIV-related proteins and TNFα [33]. Little is known about the influence of cocaine on the immune system, although some earlier studies [40] have shown a smaller stimulation of cord lymphocytes by PHA and PMA and lower levels of IL-1 and IL-2 in cord blood from newborns of drug abusing mothers.

## Conclusions

We conclude that in our study setting, uninfected infants born to HIV+ women, several immunological abnormalities could be detected. They were related to the residual maternal immune changes caused by the HIV infection as well as the use of antiretroviral drugs during pregnancy. Maternal smoking was associated to changes in cord CD3/CD4 cells, and maternal hard drug abuse was associated to more pronounced changes in the cord B cell line. Immunological changes in uninfected infants born to HIV+ women may persist for several years [2,9-11]. Little is known about their long term clinical significance. It would be interesting to examine in detail the evolution of the PB lymphocyte subsets in our study cohort. The role of maternal smoking and drug abuse on the development of the immune system of their offspring should also be studied in larger cohorts of mother-child pairs.

## Competing interests

The authors declare that they have no competing interests.

## Authors' contributions

EBA made the data collection and analysis from mothers, performed all the laboratory tests and participated in the data analysis as well as the elaboration of the manuscript. HMPMM was responsible for the selection and treatment of the HIV+ mothers, performed all the deliveries and participated in the elaboration of the draft. MMSV participated in the study design, gave support to all the immunological examinations, and revised the manuscript. FGPC and SCRA supervised the flow cytometric analysis and made its interpretation. BMA collected clinical data from the newborns, made the follow-up of the infants and participated in the data analysis. KM participated in the elaboration of the study design and made all the statistical analysis. ILM made the conception of the study, supervised all the data analysis and elaborated and made the critical revision of the manuscript.

All authors read and approved the final manuscript.

## Pre-publication history

The pre-publication history for this paper can be accessed here:

http://www.biomedcentral.com/1471-2334/11/38/prepub
